# Allergic rhinitis aggravated by air pollutants in Latin America: A systematic review^☆^

**DOI:** 10.1016/j.waojou.2021.100574

**Published:** 2021-08-20

**Authors:** Nelson A. Rosario Filho, Rogério Aranha Satoris, Wanessa Ruiz Scala

**Affiliations:** a Federal University of Parana, Curitiba, PR, Brazil; b Sanofi Pharmaceuticals, CHC Brazil Medical Affairs, Brazil

**Keywords:** Allergic rhinitis, Air pollutants, Indoor pollution, Outdoor pollution, Latin America

## Abstract

The aim of this systematic review (SR) was to evaluate the most frequent pollutants and their effect on allergic rhinitis in Latin American countries. Observational studies up to December 2020 and comparing different indoor and outdoor pollutants that had allergic rhinitis (AR) as an outcome were included in the systematic review. Random-effect meta-analyses were conducted for the presence of allergic rhinitis. Estimates were presented as pooled odds ratios (ORs) and their respective 95% confidence intervals (CIs). Twenty-two publications comprised this review according to the inclusion and exclusion criteria and 12 had data that could be analyzed statistically. The most frequent pollutant was PM10, followed by NO2 /O3 and PM2.5 in studies conducted in Argentina, Brazil, Bolivia, Chile, Colombia, Costa Rica, and Peru. The OR of an exposed subject experiencing allergic rhinitis was 1.43 (95% CI 1.026; 1.980). The OR of children and adolescents experiencing of allergic rhinitis was 1.359 (95% CI 1.051; 1.759). Asymmetry and great variability in the effect estimated from the selected studies were observed. The publication bias was quantified by Kendall's correlation and Egger's test resulted in 0.152 (p-value = 0.493). Egger's test provided an intercept equal to 2.511 and a p-value = 0.398. The I2 statistic was 89.3% and reinforces the hypothesis of heterogeneity. This first systematic review conducted in Latin America confirmed the chance of a person exposed to pollutants and experiencing allergic rhinitis is 43% greater than that of a non-exposed person, reinforcing the importance of policies to reduce pollutant exposure and the use of protection systems for workforces exposed to occupational pollutants in work environments.

## Background

Rhinitis is an inflammatory reaction of the nasal mucous membranes characterized by sneezing, nasal itching, rhinorrhea, and nasal congestion in the absence of a cold.^1^ Although sometimes mistakenly considered a trivial disease, rhinitis symptoms may significantly affect the patient's quality of life and can be associated with conditions such as fatigue, headache, cognitive impairment, and sleep disorder,^2^ in addition to affecting work performance.^3^ Allergic rhinitis (AR) affects up to 40% of the worldwide population.^4^^,^^5^

The Allergic Rhinitis and its Impact on Asthma (ARIA) group has proposed a classification for allergic rhinitis into 4 categories:^1^ mild intermittent,^2^ mild persistent,^3^ moderate/severe intermittent, and^4^ moderate/severe persistent.^6^^,^^7^

Epidemiological studies have shown that ambient air pollution is significantly associated with increased morbidity of asthma and allergic diseases.^8^ However, studies focusing on the association between long-term air pollution and rhinitis severity are scarce. European studies on respiratory health have showed that annual air pollution exposure was associated with increased severity of rhinitis, in particular for particulate matter (PM) metrics.^9^

Ocular allergy is a common comorbid condition of AR that is usually neglected and not treated accordingly. Conjunctivitis in pediatric patients has been shown significant association with the level of PM10 — particulate with a maximum diameter of 10  μm.^10^ In addition, the high prevalence of severe forms of allergic conjunctivitis, including atopic keratoconjunctivitis and vernal keratoconjunctivitis, has been significantly associated with levels of air pollutants.^11^

The mechanisms by which air pollution affects rhinitis may not be the same as for asthma according to the pollutant, as well as on the phenotype of rhinitis studied, and in particular on allergy sensitization. Air pollution is related to substances emitted above the levels allowed in ambient air. Pollutants are generated as solid (particulate), liquid, or gaseous emissions. Based on their source and derivation, these pollutants can be classified into indoor or outdoor, and primary (if directly emitted into the atmosphere) or secondary pollutants (if they react or interact therein, eg, ozone). Biological air pollution caused by aeroallergens contributes to indoor or outdoor exposure, aggravating allergic rhinitis (AR) and asthma.^12^

No systematic review has investigated the most frequent pollutants and their effect on rhinitis in Latin American countries. Thus, the aim of this systematic review was to identify the outdoor and indoor polluting agents related to allergic rhinitis in patients in Latin American countries. The following focused questions were addressed: (a) Which is the most frequent pollutant in allergic rhinitis in Latin America? and (b) What is the effect of pollution (indoor and/or outdoor) on allergic rhinitis?

## Methods

This review has followed the Preferred Reporting Items for Systematic Reviews and Meta-Analyses (PRISMA)^13^^,^^14^ and Meta-analysis of Observational Studies in Epidemiology (MOOSE) guidelines.^15^ The protocol was registered in the International Prospective Register of Systematic Reviews – PROSPERO (CRD42020211120).

### Eligibility criteria

Only observational studies (cross-sectional and longitudinal studies) were included in this systematic review. No randomized clinical trial has been identified in which allergic rhinitis was reported as an outcome. The inclusion criteria were: a) original studies published; b) data on indoor and outdoor pollutants that might be related to rhinitis; c) studies that had allergic rhinitis as an outcome. Narrative reviews, case series, case reports, *in vitro,* and animal studies were excluded. Furthermore, we excluded studies that did not have allergic rhinitis patients in the analysis, or that did not associate pollutants with allergic rhinitis or studies about pollen allergy, and even those that did not have adequate information on allergy related to indoor and outdoor polluting agents.

### Search strategy

The following databases were electronically searched: MEDLINE (PubMed), Lilacs, Embase, EbscoHost in October 2020. The following search strategy was used: 1 - rhinitis or “allergic rhinitis” or allergic or rhinosinusitis or “occupational rhinitis” or “allergic perennial” or rhinoconjunctivitis; 2 – “air pollutants” or “air pollution” or pollutants or “indoor air quality” or “traffic-related pollution;” 3 - “Latin America” or isolated countries from Latin America; 4–1 and 2 and 3. A hand search of reference lists from included publications was also conducted. In the first phase, we screened titles and abstracts identified by the search strategy. In the second phase, we screened full texts of studies meeting the inclusion criteria, or those with unclear information in the title and abstract. Rejection of studies were recorded for each report.

### Data extraction

The items extracted from publications meeting the inclusion criteria were: author, year, country, study design, setting, population characteristics, sample size, measures of exposure (pollutants), outcome measures (allergic rhinitis), results, conclusions, conflict of interest, and source of funding.

### Risk of bias

An adapted version of the Newcastle-Ottawa scale (NOS) assessed the risk of bias of cohort studies.^16^ The NOS for cohort studies comprised 10 questions about selection of the study groups, comparability of the groups, outcome, and statistical analysis. The top score was 8. Studies with 6–8 stars were arbitrarily rated as low risk of bias, 4–5 stars as moderate risk of bias, and <3 as high risk of bias.

### Summary measures and synthesis of results

Analyses of data extracted from cross-sectional studies were performed with the SAS statistical software. Random-effect meta-analyses were conducted for the presence of allergic rhinitis. The estimates were presented as pooled odds ratios (ORs) and their respective 95% confidence intervals (CIs). Cochran's Q test tested the heterogeneity and this was quantified using the I-square test. Funnel plot visual analysis, Kendall's correlation coefficient and Egger's test were used to assess publication bias.

## Results

### Search results and excluded trials

The search resulted in 382 articles: 152 from Medline, 17 from Lilacs, 165 from Embase, 0 from OpenGrey, 48 from EbscoHost. Firstly, duplicate references were excluded. From a total of 306 papers potentially relevant to this review retrieved from the electronic databases and from hand searching, titles and abstracts were evaluated based on the eligibility criteria and 214 were excluded. Of the 92 articles selected, 11 were excluded because the publication was not available and authors did not respond after 2 contact attempts, in which we requested the paper, and one Japanese article was also excluded because no other language was available. In the second phase, the texts of the remaining 80 publications were reviewed in full and, out of these, 58 were not eligible for inclusion. At the end, 22 publications^17^, ^18^, ^19^, ^20^, ^21^, ^22^, ^23^, ^24^, ^25^, ^26^, ^27^, ^28^, ^29^, ^30^, ^31^, ^32^, ^33^, ^34^, ^35^, ^36^, ^37^, ^38^ comprised this review according to the inclusion and exclusion criteria of the study. Of these, 12 had data that could be analyzed statistically,^17^^,^^18^^,^^23^^,^^24^^,^^27^, ^33^^,^^36^^,^^38^ as listed in Table 1, Table 2. The age groups in these 12 articles were as follows: children only,^4^ children and adolescents aged 5–15 years,^5^ adolescents,^1^ and adults.^2^

**Table 1:** Summary of the cross-sectional studies included in this review (n = 18)

Author, year (country)	Participants	Setting	Definition of pollutants (exposure of interest)	Definition of rhinitis	Outcomes of interest
Almeida, L. O., 2020^16^ (Brazil)	Children - students (aged 4–5 years).	Five collecting points: A, B, C, D (north of the highway 50, 450, 900, and 1500 m; the D point was just 5 m away from a municipal park) and E (450 m from the highway heading west;	Atmospheric pollutants: PM10, NO2, and O3.	International Study of Asthma and Allergies in Childhood - ISAAC Questionnaire	Prevalence of child respiratory morbidity in urban areas.
Azalim, S. P., 2014^17^ (Brazil)	Children aged 6–7 years and adolescents aged 13–14 years	Children were recruited from 13 randomly selected schools, living in the same town for at least a year, and in a household less than 2 km distant from their schools.	Concentrations of PM10 were collected in 6 out of the 13 randomly- selected schools by means of the Green Dust Monitor (Green Dust, Ainring, Germany) in a central and open spot.	ISAAC Questionnaire	Current AR, asthma and AR-asthma comorbidity.
Estévez-García, J. A., 2013^31^ (Bolivia)	Traffic police officers	Twelve operational areas in Bogotá’s metropolitan area, divided into five groups: environmental control, bus and airport terminal control, road construction group, mass transit system control and tolls on the city's limits.	Exposure to particles less than 10 μm in diameter (PM10)	American Thoracic Society's Division of Lung Disease (ATS-DLD-78 questionnaire)	Respiratory symptoms and diagnosis of respiratory alteration. Toxicological medical assessment
Goméz, M., 2009^32^ (Argentina)	Adolescents - students aged 13–14 years	23 schools in Salta, Argentina	Personal and parental smoking	ISAAC Questionnaire	Current asthma or rhinitis
Graudenz, G. S., 2002^33^ (Brazil)	Office workers	Three different office buildings, located in the downtown area of São Paulo, in the same block and belonging to the same banking company	Microbiological Assessment and dust	A combination of two questionnaires: the ATS-DLD-78,20,21 and ISAAC	Prevalence of respiratory symptoms among a group of office workers in São Paulo
Herrera, R., 2017^34^ (Chile)	Chilean children living close to the mines	Estimating the Causal Impact of Proximity to Gold and Copper Mines	TSP (total suspended particulate) and PM10 pollution.	ISAAC Questionnaire	Asthma or allergic rhinoconjunctivitis.
Hüttner, M. D., 2000^35^ (Brazil)	Workers of the fertilizer industry	Rio Grande, RS	Agents present in the nitrogen fertilizer production industry, the presence of free silica, gaseous fluorides and ammonia, at concentrations above tolerance limits.	American Thoracic Society's Division of Lung Disease (ATS-DLD-78 questionnaire)	Clinical, radiological manifestations and respiratory function of exposed employees.
Miño, L. A., 2013^38^ (Colombia)	Boys and girls aged 6–14 years.	4 zones with different levels of contamination of the city of Santa Marta, Colombia.	PM10	ISAAC Questionnaire	Respiratory symptoms and lung function disorder.
Nicolussi, F. H., 2014^37^ (Brazil)	Schoolchildren aged six to seven years	Schools	Concentrations of inhaled particles (PM10), nitrogen dioxide (NO2), ozone (O3), and relative humidity (RH%)	ISAAC Questionnaire	Prevalence of allergic respiratory diseases.
Riguera, D., 2011^18^ (Brazil)	Schoolchildren	Monte Aprazível, Southeastern Brazil	Environmental pollution from burning sugarcane straw: fine particulate matter (PM2.5) and Black carbon	ISAAC Questionnaire	Prevalence of respiratory symptoms and peak expiratory flow measurements in schoolchildren: PEF = Peak Expiratory Flow, PM2.5 concentration and rhinitis prevalence
Robinson, C. L., 2011^19^ (Peru)	Adolescents aged 13 and 15 years	170 households	PM2.5 concentrations (mg/m3)	ISAAC Questionnaire	Asthma symptoms, allergy and airway inflammation
Rodríguez-Moreno, N., 2013^20^ (Colombia)	Children aged 5–14 years	An area of Bogota, 2012–2013	Emissions from chimneys of industries distributed disorderly in local territory, scattered recycling areas, burning wood for coal generation, burning tires, and foul odors arising from these activities, along with those of contaminated water sources and sewerage that occasionally overflows in some neighborhoods, likewise a large percentage of damaged road mesh	ISAAC Questionnaire	Prevalence of respiratory symptoms, asthma and rhinitis.
Rodríguez-Zamora, M. G., 2018^21^ (Costa Rica)	Workers from grain storage facilities (operators and administrative staff and other workers)	Grain Storage Facilities in Costa Rica	Grain dust	European Community Respiratory Health Survey questionnaire	Self-reported respiratory health outcomes, rhinitis, and eczema.
Sih T., 1999^23^ (Brazil)	Children (age range between 7 and 14 years)	São Paulo city and a rural area around the city of Tupã	Urban air pollution	Standardized questionnaire applied by a physician to evaluate positive symptoms of nose itching, sneezing episodes and perennial clear nose secretion	Children's respiratory health.
Solé, D., 2007^24^ (Brazil)	Adolescents (aged 13–14 years)	Brazilian cities with a high population index and high air pollution levels.	Photochemical air pollutants (O3, NO2, SO2, and CO)	ISAAC Questionnaire	Prevalence of symptoms of asthma, rhinitis and atopic eczema in adolescents
Solis-Soto, M.T., 2013^25^ (Bolivia)	Children attending the fifth grade in elementary schools	From the Oropeza Province – Bolivia:	Outdoor and indoor pollutants	ISAAC Questionnaire	Asthma, rhinoconjunctivitis and eczema symptoms
Souza, R.M., 2010^26^ (Brazil)	Individuals who worked directly in the production of charcoal and who were registered as charcoal workers	Cities of Lindolfo Collor, Ivoti and Presidente Lucena, Brazil	Charcoal pollution	Allergic rhinitis defined as the presence of nasal itching, nasal obstruction and nasal secretion	Prevalence of respiratory symptoms and smoking, as well as lung function parameters
Toledo, M.F., 2016^27^ (Brazil)	Adolescents aged 13–14 years attending public and private schools	Taubaté, São Paulo, Brazil (2005–2012):	Urban air pollution	ISAAC Questionnaire	Prevalence of asthma, rhinitis, and eczema

**Table 2:** Summary of the longitudinal studies included in this review (n = 4)

Author, year (country)	Participants	Setting	Definition of pollutants (exposure of interest)	Definition of rhinitis	Outcomes of interest
Bose, S., 2018^28^ (Lima)	Children aged 9–19 years	Fifteen outdoor locations from each community (n = 30 total) Pampas de San Juan de Miraflores (Pampas); Villa el Salvador (Villa);	Particulate matter < 2.5 μm, defined as week-long ambient concentrations of PM2.5 (particulate matter with aerodynamic diameter < 2.5 μm), black carbon (BC), temperature, and humidity	ISAAC Questionnaire	RQLQ (Rhinoconjunctivitis Quality of Life Questionnaire), which was categorized as not bothered (score = 0) or bothered (score > 0)
Sánchez, J., 2018^22^ (Colombia)	Children (aged 6–14 years) with a diagnosis of asthma or rhinitis living for more than five years in urban or rural areas	Antioquia - Colombia.	Urban Pollution;	Allergic Rhinitis Symptom Questionnaire (ARSQ) to evaluate the severity of rhinitis - the 7 most common symptoms of rhinitis, rated on a scale of 0–4 points, are assessed according to intensity (Absent to very severe).	Severity and treatment of asthma and rhinitis in terms of the dose required for control. Evolution of patients over time focused on treatment response and severity of respiratory diseases
Trevisan, I·B., 2018^29^ (Brazil)	Male sugarcane workers	Brazil	Exposure to fine particulate matter PM2.5 (μg/m3) and climate variations for Temperature (°C) and relative humidity (%), during: Non-harvesting (NHP - sugarcane plantation, when there is no burnt sugarcane harvesting), and Harvesting (HP - 3 and 6 months after the beginning of harvesting)	Rhinitis is defined as the presence of nasal congestion, rhinorrhea, sneezing and/or nasal itching.	Rhinitis symptoms and inflammatory markers in sugarcane workers.
Wichmann, F.A., 2009^30^ (Argentina)	Children aged 6–12 years living close to the petrochemical plants in La Plata, Argentina	Study locations in La Plata, Argentina. Four locations: 4 specific areas were chosen for the study: “industrial”, next to the petrochemical complex;	Particulate matter (PM) and Volatile organic compounds (VOCs)	ISAAC Questionnaire	Respiratory health of children aged 6–12 years.

**Fig. 1:**
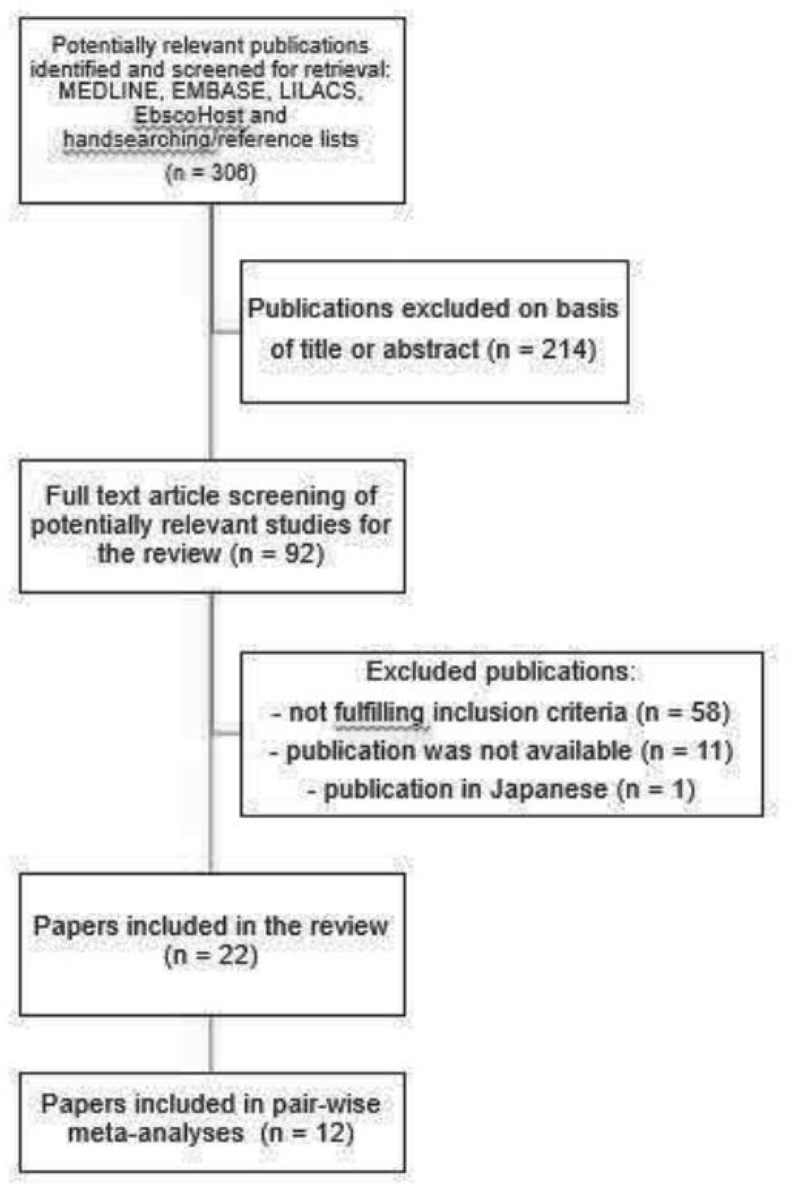
Flowchart of the publications searched in this review

Cross-sectional and longitudinal studies were only reviewed if related to allergic rhinitis, rhinosinusitis, occupational rhinitis, perennial allergic rhinitis, and rhinoconjunctivitis in association with pollutants as outcome (Fig. 1).

### Studies included-

#### Cross-sectional studies

Eighteen cross-sectional studies remained in this review.^17^^,^^18^ Their characteristics are presented in Table 1. A total of 31 205 subjects from both sexes, aged between 4 and 59 years, were included. More than half of the studies were conducted in Brazil, 2 in Bolivia, 2 in Colombia, 1 in Argentina, 1 in Chile, 1 in Peru, and 1 in Costa Rica. For allergic rhinitis assessment, self-administered questionnaires and interviews were employed. An important point verified is that in most papers included in this systematic review a doctor was not involved in allergic rhinitis diagnosis.

Most of the included studies verified outdoor pollutants often vehicle emissions, such as carbon monoxide (CO), sulfur dioxide (SO2), nitric oxide (NO), nitrogen dioxide (NO2), and particulate matter (PM10), which encompasses a mixture of organic and inorganic substances, such as heavy metals, hydrocarbons, and microorganisms.^39^ The most frequent pollutant was PM10 (6 studies), followed by NO2 /O3 (3 studies) and PM2.5 (2 studies).

#### Longitudinal studies

Among the 22 reviewed studies, 4^21^, ^22^, ^23^^,^^35^ were longitudinal studies conducted in Argentina, Brazil, Colombia, and Peru. Their characteristics are shown in Table 2. A total of 2094 individuals, aged 6–48 years, were included in those studies. In 3 studies,^21^^,^^23^^,^^35^ subjects were ≤19 years of age and, in 1,^22^ subjects were adult males. Allergic rhinitis was identified by means of self-reported questionnaires, whereas exposure to pollutants was assessed by equipment installed in the environment of interest. The most frequent pollutant was PM2.5 (2 studies).

### Methodological quality of the included studies

#### Cross-sectional studies

The risk of bias of the cross-sectional studies was assessed according to the NOS domains (Table 3). Of the 18 cross-sectional studies included, 9 (50%) were considered to have a low risk of bias,^17^^,^^18^^,^^20^^,^^24^^,^^28^^,^^30^^,^^32^^,^^33^^,^^36^ eight (44.4%) presented a moderate risk,^19^^,^^25^, ^26^, ^27^^,^^31^^,^^34^^,^^37^^,^^38^ and 1 (5.6%)^29^ had a high risk of bias.

**Table 3:** Risk of bias assessment of the included cross-sectional studies

Author, year	Selection (maximum 4)	Comparability (maximum 1)	Exposure /Outcome (maximum 3)	Total (maximum 8)
Almeida, L. O., 2020^17^	3	1	3	7 Low
Azalim, S. P., 2014^18^	3	1	2	6 Low
Estévez-García, J. A., 2013^24^	3	1	2	6 Low
Goméz, M., 2009^25^	3	1	1	5 Moderate
Graudenz, G. S., 2002^26^	3	1	1	5 Moderate
Herrera, R., 2017^27^	3	1	1	5 Moderate
Hüttner, M. D., 2000^28^	3	1	2	6 Low
Miño, L. A., 2013^30^	3	1	2	6 Low
Nicolussi, F. H., 2014^31^	3	0	2	5 Moderate
Riguera, D., 2011^29^	1	0	1	2 High
Robinson, C. L., 2011^32^	3	1	3	7 Low
Rodríguez-Moreno, N., 2013^33^	3	1	2	6 Low
Rodríguez-Zamora, M. G., 2018^34^	2	1	2	5 Moderate
Sih, T., 1990^36^	3	1	2	6 Low
Solé, D., 2007^37^	3	0	1	4 Moderate
Solis-Soto, M. T., 2013^38^	3	1	1	5 Moderate
Souza, R. M., 2010^19^	2	1	1	4 Moderate
Toledo, M. F., 2016^20^	3	1	2	6 Low
Author, year	Selection (maximum 4)	Comparability (maximum 1)	Exposure /Outcome (maximum 3)	Total (maximum 8)
Bose, S., 2018^21^	4	1	2	7 Low
Sánchez, J., 2018^35^	3	1	2	6 Low
Trevisan, I·B., 2018^22^	3	1	2	6 Low
Wichmann, F.A., 2009^23^	2	1	2	5 moderate

#### Longitudinal studies

The risk of bias of longitudinal studies is shown in Table 4. None of the studies were considered to have a high risk of bias. In fact, most studies^21^^,^^22^^,^^35^ had a low risk of bias and only 1 study^23^ presented a moderate risk of bias.

**Table 4:** Risk of bias assessment of the included longitudinal studies

Author, year	Selection (maximum 4)	Comparability (maximum 1)	Exposure /Outcome (maximum 3)	Total (maximum 8)
Bose, S., 2018^21^	4	1	2	7 low
Sánchez, J., 2018^35^	3	1	2	6 low
Trevisan, I·B., 2018^22^	3	1	2	6 Low
Wichmann, F.A., 2009^23^	2	1	2	5 moderate

A total of 32 873 individuals were included in the selected studies. Most of the participants were children or adolescents; only 4.94% were adults. The studies included male and female subjects, except for 3^22^^,^^28^^,^^34^ conducted exclusively in males.

### Odds ratio (OR) analysis

**Fig. 2:**
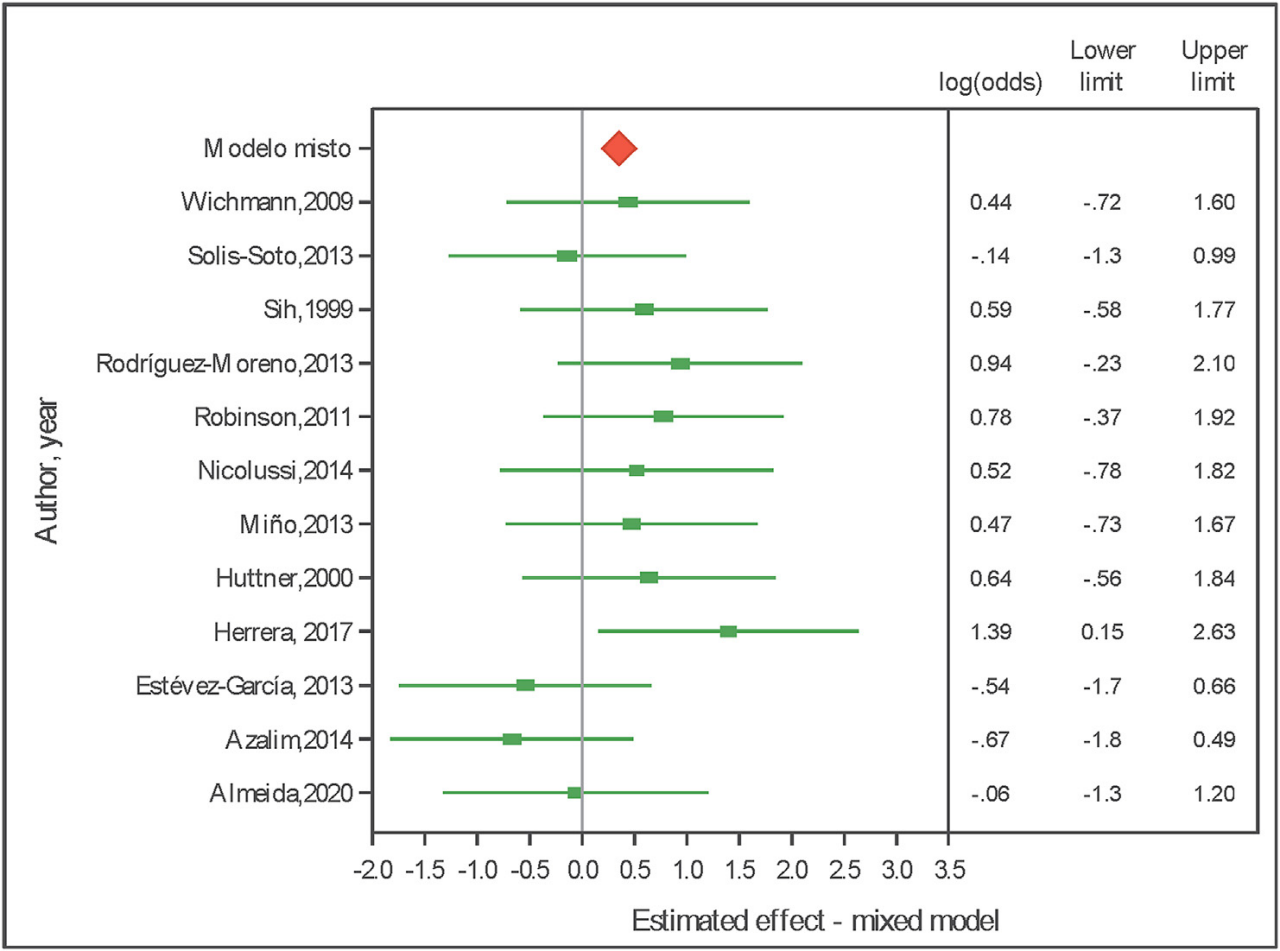
Logarithm of the odds ratio estimates (ln OR) of an exposed subject experiencing allergic rhinitis (0.355; 95% Confidence Interval 0.026–0.683). Forest plot - Mixed model

Considering the mixed model, the estimate of the natural logarithm of the odds ratio estimates (ln OR) of an exposed subject experiencing allergic rhinitis was 0.355 (95% Confidence Interval 0.026–0.683) (Fig. 2). According to the original scale, obtained from inverse transformation, the OR of an exposed subject experiencing allergic rhinitis was 1.43 (95% Confidence Interval 1.026–1.980), ie, according to the model, the chance of a person exposed to pollutants of experiencing rhinitis was 43% greater than that of a non-exposed person.

### Children and adolescents

**Fig. 3:**
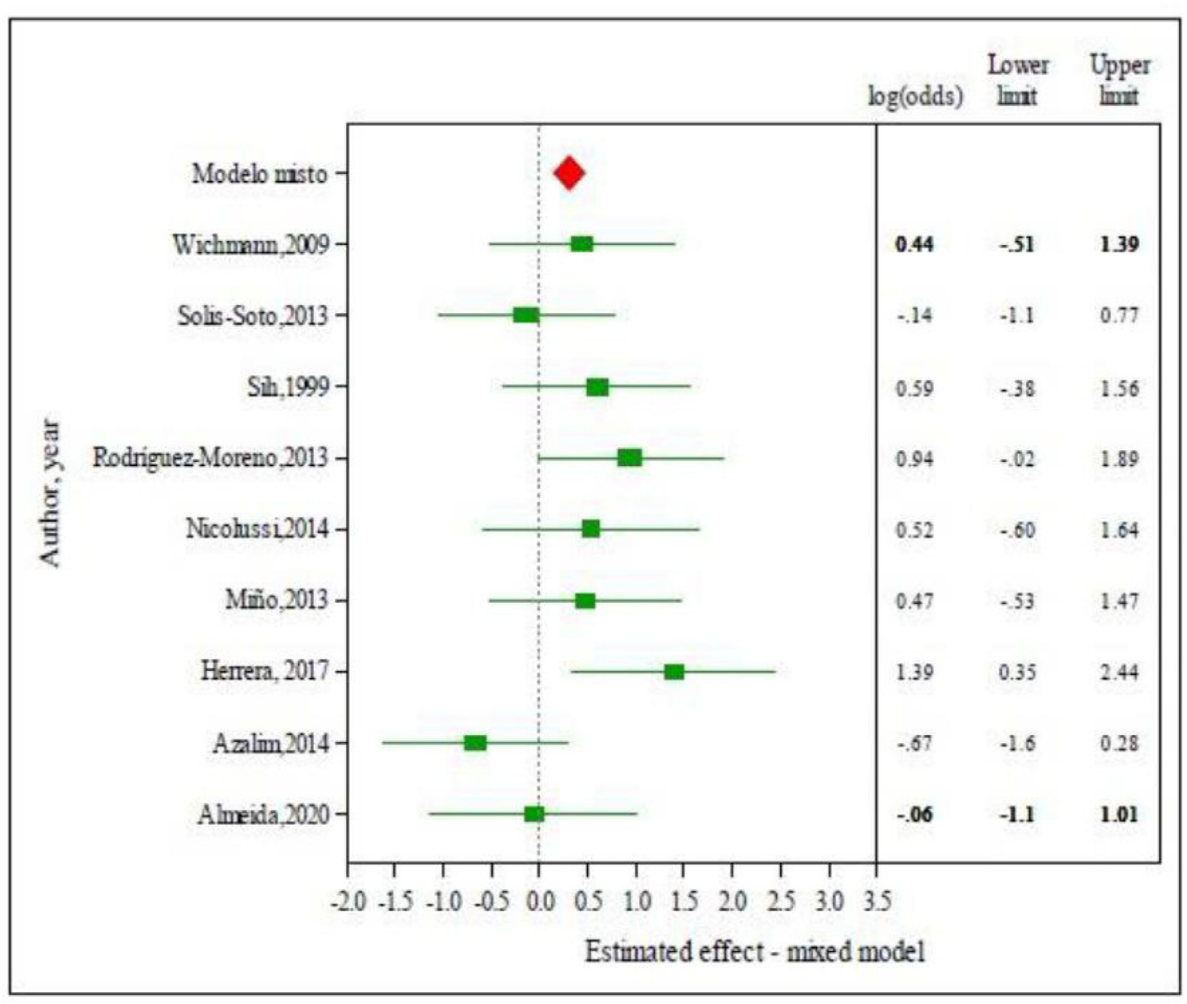
Logarithm of the odds ratio value (ln OR) of children and adolescents experiencing allergic rhinitis (OR 0.307; 95% Confidence Interval 0.050–0.565). Forest plot - Mixed model analysis

According to the mixed model, the estimate of the natural logarithm of the odds ratio value (ln OR) of children and adolescents experiencing allergic rhinitis was 0.307 (95% Confidence Interval 0.050–0.565) (Fig. 3). According to the original scale, obtained from inverse transformation, the OR of allergic rhinitis was 1.36 (95% Confidence Interval 1.051; 1.759), ie, the chance of a child /adolescent exposed to pollutants of experiencing rhinitis was 36% greater than that of a non-exposed subject.

### Publication bias

Asymmetry and great variability in the effect estimated from the selected studies were observed. The studies by Almeida,^17^ Azalim,^18^ Estévez-Garcia,^24^ and Solis-Soto^38^, presented negative effect estimates and are asymmetric in relation to positive effects. The publication bias was quantified by Kendall's correlation and Egger's test. Kendall's correlation coefficient resulted in 0.152 (p = 0.493). Egger's test provided an intercept equal to 2.511 and a p value = 0.398. This test has little power, especially when the number of studies is less than 10, therefore, non-rejection of the null hypothesis must be carefully evaluated. None of the tests showed evidence of an association between the estimated effect and its variance.

### Heterogeneity test

The value of Cochran's Q statistic, equal to 103.270 (p-value < 0.001), rejects the hypothesis of homogeneity. The I2 statistic was 89.3% and reinforces the hypothesis of heterogeneity.

## Discussion

Although air pollution is a worldwide problem, diseases attributable to particulate matter in the air predominate in low- and middle-income countries, which have the highest levels of exposure to household air pollution. Poverty limits access to information, treatment and other healthcare resources contributing to the effects of air pollution on health.^40^

Exposure to allergens is a known risk factor for the development of AR and more severe phenotypes. Experimental studies on specific pollutant exposure and controlled allergen exposure in challenge chamber suggest that pollution can exacerbate allergic airway disease and increase nasal responsiveness.^41^

Anthropogenic outdoor air pollution contributes to global warming and has a direct negative effect on human health, but also enhances the pollinating season and the allergenicity of some pollens.^42^

This meta-analysis provides available evidence that air pollution is a risk factor for AR in Latin American countries, supporting what has been demonstrated in other regions. It is important to emphasize that although the objective was to evaluate indoor and outdoor pollutants, most articles included in this systematic review involved outdoor pollution.

Besides common outdoor pollutants, such as vehicle fuel burning products, urban pollution, and indoor ventilation system particulate matter, this systematic review also included a wide variety of polluting factors and characteristic aspects of the region, such as charcoal pollution, gold and copper mine pollutants, fertilizer industry pollutants, and grain storage facilities’ effect on exposed workers, petrochemical pollution, as well as sugarcane burning products. The diversity of factors adds rich regional data to the review, but also great heterogeneity in the analysis.

Children are particularly susceptible to the health effects of air pollution. A publication of the World Health Organization (WHO) shows that air pollution has a major impact on health and survival of children. The negative effects of climate change are generally widespread, 1 in 4 deaths of children under the age of 5 is directly or indirectly related to environmental risks and many children live in environments with levels of air pollution above the WHO recommendation.^43^^,^^44^

Peculiarities that significantly affect children by the improper environment are due to biological immaturity, prenatal and postnatal lung development, higher energy, and metabolic consumption. Social behavior, such as crawling, bringing objects to their mouths, becoming more exposed to potential contaminants of dust, soil, toy components, or household cleaning products, and having no decision-making abilities on environmental issues all impact children's health.^43^^,^^45^ Epidemiological studies suggest that both indoor and outdoor air pollution increases the risk of respiratory tract infections in the pediatric population.^45^

In this review, assessing children and adolescents separately from adults, we have not found a greater impact of pollutants on that younger population. This can be explained by exposure intensity, as the adult population consisted of employees directly exposed to polluting agents at work, while studies involving children evaluated urban pollution in general.

Exposure to oxidant air pollutants (O3 and NO2), but not PM2.5, were associated with an increased risk of asthma (17%) and eczema (7%) in children as demonstrated in a longitudinal study in Canada. In multi-pollutant models, exposures to NO2 and O3 in the first 3 years of life showed a similar trend of increasing the incidence of asthma, AR, and eczema.^46^

Diesel exhaust particles increased the number of human nasal epithelial cells infected with the Influenza A virus *in vitro* through the enhancement of virus attachment and entry into respiratory cells facilitated by radical oxygen species.^47^^,^^48^

The airway epithelium is a physical barrier, which protects submucosal tissues from harmful substances. This mechanical barrier function is determined by the integrity of intercellular junctions, which consist of the apical tight junctions and underlying adherent junctions. Nasal epithelial cells exposed i*n vitro* to PM2.5 may lose the barrier effect through decreased expression of junction proteins and increased release of cytokines IL-8, TIMP metallopeptidase inhibitor 1 and TSLP with proinflammatory action. The end result could be an increased susceptibility to rhinitis and rhinosinusitis in highly PM2.5 polluted areas.^49^^,^^50^

This review should be interpreted with caution, as the results were derived from different populations and there was considerable heterogeneity in the meta-analysis. Furthermore, some studies presented a moderate risk of bias.

## Conclusions

This is the first systematic review conducted in Latin America to assess the effect of indoor and outdoor pollution on allergic rhinitis symptoms in pediatric and adult populations. The study confirmed that the chance of a person exposed to pollutants of experiencing rhinitis is 43% greater than that of a non-exposed person, reinforcing the importance of policies to reduce pollutant exposure and the use of protection systems for workforces exposed to occupational pollutants in the work environment.

## Abbreviations

AR- Allergic Rhinitis, CI: Confidence Interval, OR: odds ratio, ARIA: Allergic Rhinitis and its Impact on Asthma, WHO: World Health Organization, TSLP: thymic stromal lymphopoietin, PM: particulate matter, PRISMA: Preferred Reporting Items for Systematic Reviews and Meta-Analyses, MOOSE: Meta-analysis of Observational Studies in Epidemiology, PROSPERO: International Prospective Register of Systematic Reviews, NOS: Newcastle-Ottawa scale, CO: carbon monoxide, SO2: sulfur dioxide, NO: nitrogen oxide, NO2: nitrogen dioxide, O3: ozone, ISAAC: International Study for Asthma and Allergies in Childhood, TSP: total suspended particulate.

## Conflict of interest

Dr. Rosario Filho reports personal fees from Sanofi, during the conduct of the study; personal fees from Mylan, personal fees from AstraZeneca, personal fees from Chiesi, personal fees from Abbott, outside the submitted work.Dr.Sartoris; Medical Manager (Brazil) at Sanofi.Dr.Ruiz: Medical Head (Brazil) at Sanofi (Consumer Health Care Business Unit). (Consumer Health Care Business Unit).
